
JOURNAL INFORMATION
==============================
NLM Title Abbreviation: ScientificWorldJournal
Journal ID: ScientificWorldJournal
NLM Journal ID: 101131163
Journal ID: TSWJ

The Scientific World Journal

ISSN: 2356-6140
EISSN: 1537-744X
Publisher: Wiley

ARTICLE INFORMATION
==============================
PMCID: PMC6084644
PMID: 30147520
DOI: 10.1100/tsw.2001.170
Article ID: 721050
Article version: 1
Subjects: Short Report

Mitochondrial DNA Mutations Cause Disease, Apoptosis, and Permeability Transition Pore Dysfunction

Justin L. Mott *
Dekui Zhang
Zassenhaus H. Peter
Department of Molecular Microbiology and Immunology, St. Louis University, 1402 S. Grand Blvd., St. Louis, MO 63104, USA
*L. Mott Justin: mottjl@slu.edu
Publication date: 2001
Electronic publication date: 2001 Oct 30
Volume: 1
First page: 55
Last page: 55
Copyright: Copyright © 2001 L. Mott Justin et al.
Copyright year: 2001
License: This is an open access article distributed under the Creative Commons Attribution License, which permits unrestricted use, distribution, and reproduction in any medium, provided the original work is properly cited.
License URL: https://creativecommons.org/licenses/by/3.0/

Funding: American Heart Association American Diabetes Association National Heart, Lung, and Blood Institute

==============================
Miami *Nature Biotechnology* Short Reports
*TheScientificWorld* (2001) 1(S3), 55SR
ISSN 1532-2246; DOI 10.1100/tsw.2001.170

### MITOCHONDRIAL DNA MUTATIONS CAUSE DISEASE, APOPTOSIS, AND PERMEABILITY TRANSITION PORE DYSFUNCTION

Justin L. Mott*, Dekui Zhang, and H. Peter Zassenhaus

Department of Molecular Microbiology and Immunology, St. Louis University,
1402 S. Grand Blvd., St. Louis, MO 63104.
* mottjl@slu.edu

**INTRODUCTION.** Mitochondrial DNA (mtDNA) mutations accumulate with age (1), but are normally below the level of 1%. It is unlikely that respiratory deficiencies result from such low frequencies; indeed, we recently showed that respiratory function was preserved in mice with somatic mtDNA mutations in the heart, despite induction of severe cardiomyopathy (2). Therefore, mutations may need to invoke a dominant mechanism of dysfunction. We showed that one such mechanism, oxidative stress, was not the cause of the myopathy (3). Here we describe a second dominant mechanism, the activation of apoptosis, cytochrome c release, and permeability transition pore dysfunction. We propose that malfolded mutant mitochondrial proteins alter mitochondrial permeability by interacting with cyclophilin D, a bi-functional protein that acts as a protein-folding catalyst and as a regulator of the permeability transition pore.

**METHODS.** *Apoptosis:* Cell death was seen by H&E staining, and defined as apoptosis by nuclear morphology, lack of inflammation, and TUNEL staining. *Pore function:* The membrane potential of isolated cardiac mitochondria was monitored with JC-1 fluorescence. Pore opening was induced by calcium (500-2000 nmol/mg); pore opening was inhibitable with cyclosporin A and ADP. *Cytochrome c:* Cytoplasmic *cyt c* was assayed in S-100 supernatants by blotting. *Cyclosporin A:* CsA (50 mg/kg/day) was given orally in cremophore:olive oil.

**RESULTS.** By the time the frequency of point mutations had climbed to ~1/10,000 bases, all mice had dilated, fibrotic hearts. Increased apoptosis preceded signs of disease by about 1 week. Consistent with a mitochondrial role in cell death, *cyt c* was released from mitochondria, beginning at the same time as the onset of increased apoptosis. Given the potential role of the permeability transition pore in cell death and *cyt c* release (4), we assessed pore function in isolated heart mitochondria.

Mitochondria from mice with mtDNA mutations exhibited perturbed pore opening caused by low calcium, and even with maximal pore induction (100 μM $Ca^{++}$) retained their *cyt c*, unlike mitochondria from mice without mutations (Fig. 1; other figures appear at end of paper). The disturbed pore function and cyt c release suggest a striking change in the mitochondrial permeability properties, possibly mediated by cyclophilin D. The dual roles of cyclophilin D as a peptidyl-prolyl isomerase and a pore regulator (4) uniquely enable this protein to act as a detector of malfolded mitochondrial proteins; indeed, cardiomyopathy was prevented by treatment with the cyclophilin inhibitor cyclosporin A.

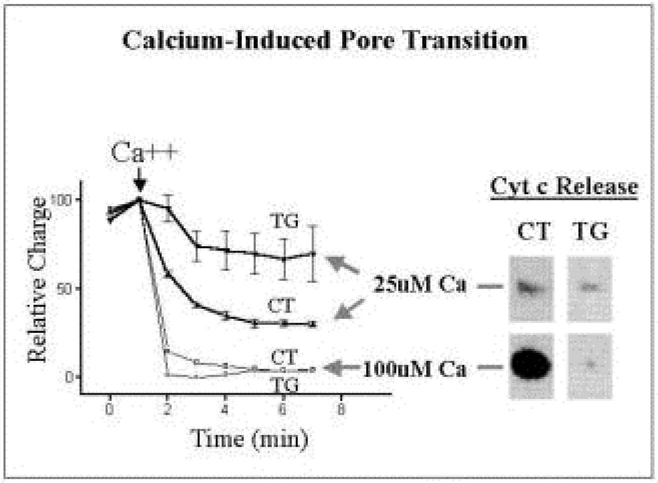

**Fig. 1: Altered Permeability of Mitochondria with mtDNA Mutations.** Mitochondrial permeability transition pore function is altered in mitochondria with mtDNA mutations (TG) compared to mitochondria without (CT). Calcium at low concentration induced less pore opening in TG mito. Pore opening was measured by loss of the membrane potential (Relative Charge), and was inhibited by cyclosporin A plus ADP (not shown). At higher [Ca++], mitochondria with and without mutations depolarized to a similar extent, but mutations still caused dysfunction, seen as retention of cytochrome c. The loss of a membrane potential while retaining cyt c suggests that pore opening did not result in swelling sufficient to cause outer membrane rupture, perhaps indicating low conductance pore opening.

**SUMMARY.** Apoptotic signaling, including opening of the mitochondrial permeability transition pore, is altered in mice with mtDNA mutations. Further, the disease that follows can be prevented by CsA, an inhibitor of pore opening and apoptosis. We hypothesize that cyclophilin D acts as a detector of mutations and signals the cell through altered pore function. Initiation of apoptosis is a dominant mechanism whereby rare mutations can result in disease.

**ACKNOWLEDGEMENTS.** Supported by grants from the American Heart Association, the American Diabetes Association, and the NIH Aging and Heart, Lung, & Blood Institutes.

**REFERENCES.**

1. Wallace, D.C. (1999) Science 283, 1482-1488
2. Zhang, D., Mott, J.L., Chang, S-W., Denniger, G., Feng, Z., Zassenhaus, H.P. (2000) Genomics 69, 151-161

3. Mott, J.L., Zhang, D., Stevens, M., Chang, S-W., Denniger, G., Zassenhaus, H.P. Mutat. Res. (in press)
4. Halestrap, A.P. (1999) Biochem. Soc. Symp. 66, 181-203

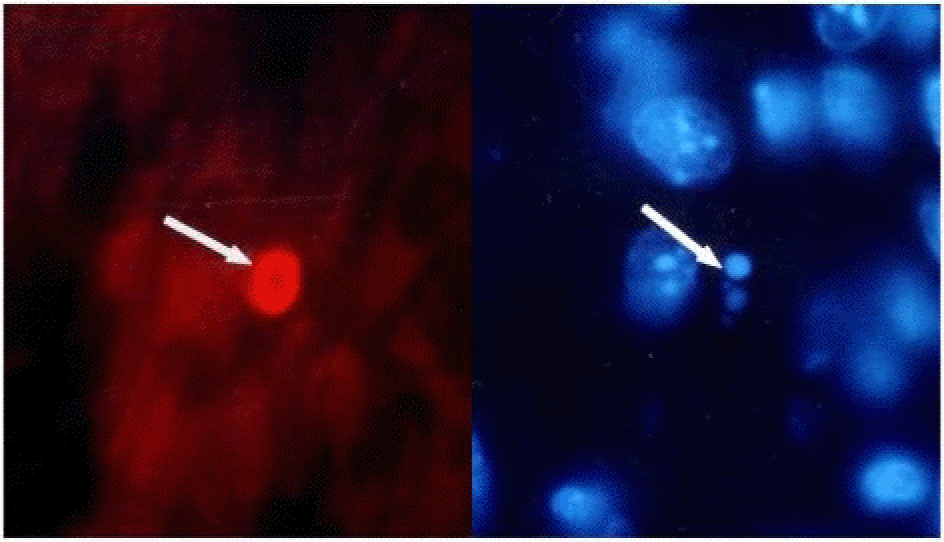

**Fig. 2: Mitochondrial DNA Mutations Cause Apoptosis.** Hearts with mtDNA mutations have higher numbers of apoptotic cells. TUNEL staining (left panel; rhodamine detection system- red) revealed higher numbers of apoptotic myocytes in hearts with mitochondrial mutations than without mutations. Other features were used to support an apoptotic mechanism of cell death including nuclear fragmentation and contraction by DAPI staining (right panel; blue staining), and the lack of inflamation (not shown).

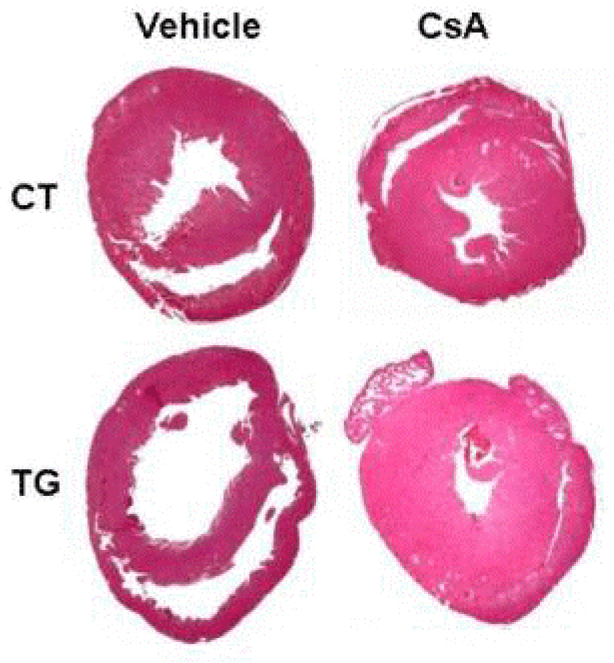

**Fig. 3: Inhibition of Pore Opening Prevents Disease.** Mice with mtDNA mutations in the heart (TG) developed congestive heart failure and cardiac dilation (cf. Vehicle TG with Vehicle CT). Heart disease may depend on dysfunction of the permeability transition pore, as treatment with cyclosporin A (inhibitor of pore opening) prevented dilation (CsA TG). CsA inhibits pore opening via matrix cyclophilin D. We propose that cyclophilin D acts as a detector of mtDNA mutations.
